# Evaluation of cerebrospinal fluid treponema pallidum particle agglutination assay titer for neurosyphilis diagnosis among HIV-negative syphilis patients

**DOI:** 10.3389/fimmu.2025.1572137

**Published:** 2025-03-28

**Authors:** Mei Shi, Fuquan Long, Danyang Zou, Xin Gu, Liyan Ni, Yuanyuan Cheng, Tengfei Qi, Wei Zhao, Lin Zhu, Zhifang Guan, Pingyu Zhou

**Affiliations:** Department of Sexually Transmitted Disease, Shanghai Skin Disease Hospital, School of Medicine, Tongji University, Shanghai, China

**Keywords:** neurosyphilis, immunodiagnosis, treponemal test, cerebrospinal fluid, Treponema pallidum

## Abstract

**Purpose:**

The study aimed to assess the association between CSF-TPPA titer and the presence of neurosyphilis, and to determine the optimal CSF-TPPA titer cutoff for diagnosing neurosyphilis.

**Methods:**

We conducted a cross-sectional study at a single center from April 2020 and January 2022.Receiver operating characteristic (ROC) analysis was used to assess the performance of CSF-TPPA titer in the diagnosis for neurosyphilis. The relationship between CSF-TPPA titer and neurosyphilis was investigated by restricted cubic spline (RCS) curves.

**Results:**

A total of 715 HIV-negative syphilis patients were included in the final analysis. CSF-TPPA was reactive in 443 cases (62.0%), with a median titer of 1:160 (IQR, Negative to 1:2560). The area under curve (AUC) of CSF-TPPA titer was 0.967 (95% Confidence interval (CI): 0.951- 0.979). CSF-TPPA titer ≥ 1:320 provided a sensitivity of 92.53% and a specificity of 87.96% for the identification of neurosyphilis. For those presumptive neurosyphilis patients, CSF-TPPA ≥1:320 could effectively identified symptomatic neurosyphilis. The RCS curve revealed a non-linear and positive association between CSF-TPPA titer and risk of neurosyphilis. After full adjustments for confounding covariates, it showed that the prevalence of neurosyphilis was relatively flat until CSF-TPPA titer reached 1:320 and then started to escalated rapidly afterwards.

**Conclusion:**

This study revealed that a CSF-TPPA titer ≥ 1:320 can be used as a highly sensitive and practical marker for diagnosing neurosyphilis. A titer threshold of ≥1:320 could offer a reliable alternative in cases when CSF-VDRL is negative.

## 1 Introduction

Neurosyphilis is a serious manifestation of syphilis that can affect the central nervous system and lead to irreversible neurological damage if not diagnosed and treated promptly. The diagnosis of neurosyphilis remains challenging due to the limitations of existing laboratory tests. Currently, the cerebrospinal fluid venereal disease research laboratory (CSF-VDRL) test is considered the gold standard for confirming neurosyphilis (1, 2). However, CSF-VDRL exhibits low sensitivity (3, 4), particularly in early-stage disease, resulting in false-negative results (5, 6). In such cases, elevated cerebrospinal fluid (CSF) white blood cell count (WBC) and/or protein levels, in conjunction with a positive Treponema pallidum particle agglutination (TPPA) test, are often considered highly suggestive of neurosyphilis (1, 2, 7).

Historically, CSF tests for treponema pallidum specific antibody, including TPPA, were thought to possess high sensitivity but low specificity, making them unreliable for confirming neurosyphilis (8). Nevertheless, a negative TPPA could exclude the diagnosis of neurosyphilis, especially when CSF-VDRL was also negative. In recent years, it has been proposed that semiquantitative titration of CSF-TPPA could improve the diagnostic specificity for neurosyphilis. Specifically, a higher CSF-TPPA titer cutoff has been suggested to enhance the accuracy of diagnosis (9–11). The UK national guidelines (12) recommend a cutoff of > 1:320 for CSF-TPPA titers in diagnosing neurosyphilis. However, this approach has not been widely incorporated into many major clinical guidelines, and its role remains under evaluation.

The objective of this study was to assess the association between CSF-TPPA titer and the presence of neurosyphilis, and to determine the optimal CSF-TPPA titer cutoff for diagnosing neurosyphilis. We aim to evaluate these parameters within a large cohort of HIV-negative syphilis patients to establish more precise diagnostic criteria for this challenging condition.

## 2 Methods

### 2.1 Study population and data collection

This cross-sectional study was conducted at the sexually transmitted disease (STD) department of the Shanghai Skin Disease Hospital, School of Medicine, Tongji University from April 2020 to January 2022. Patients included in the study underwent lumbar puncture (LP) for the screening neurosyphilis and met one of the following criteria 1) syphilis at any stage without prior treatment; 2) after receiving initial recommended treatment and at least 12 months of regular follow-up, no 4-fold decrease or negative conversion in serum toluidine red unheated serum test (TRUST) titer; or 3) exhibiting neuropsychiatric, ocular, or otic symptoms. Patients who had been previously diagnosed with or treated for neurosyphilis were excluded from the study. The diagnosis of neurosyphilis or non-neurosyphilis was based on clinical assessment and applied to HIV-negative syphilis patients. Exclusion criteria included pregnant or lactating women, patients who had failed LP procedure, those with incomplete clinical data, and those with CSF containing blood.

All eligible patients who agreed to participate provided written informed consent. Participants were interviewed with a brief questionnaire to collect medical and socio-demographic information prior to undergoing LP. The study protocol was approved by the Medical Ethics Committee of the Shanghai Skin Disease Hospital (Ethics number: 2020-16) and conducted according to the principles expressed in the Declaration of Helsinki.

### 2.2 Case definitions

The confirmed laboratory diagnostic criteria for neurosyphilis in syphilis patients included a reactive cerebrospinal fluid-venereal disease research laboratory (CSF-VDRL) test. The presumptive neurosyphilis occurs when the CSF-VDRL is negative, but the CSF protein concentration exceeds 0.5g/L and/or the CSF white blood cell count is greater than 10/μL, with no other known cause for these abnormalities (13).

Symptomatic neurosyphilis was defined as the presence of CSF abnormalities the above mentioned consistent with neurosyphilis, accompanied by neuropsychiatric signs or symptoms. Otherwise, it is defined as asymptomatic neurosyphilis (1). Each criterion for symptomatic neurosyphilis were classified according to the STI guidelines of the United States Centers and Chinses for Disease Control and Prevention (CDC) and European (1, 2, 7). The classification diagnosis for symptomatic neurosyphilis includes the following categories: general paresis (e.g., mental and behavioral abnormalities, cognitive dysfunction), tabes dorsalis (e.g., sensory ataxia, Adie’s pupil), meningovascular syphilis (e.g., symptoms or signs of stroke), meningeal syphilis (e.g., symptoms or signs of meningitis), ocular syphilis (e.g., vision loss, double vision) and otosyphilis (e.g., hearing loss, deafness).

### 2.3 Specimen collection and laboratory tests

Serum specimens were screened using the TRUST test (Rongsheng Biotech Co., Ltd, Shanghai, China) and confirmed with the T. pallidum particle agglutination (TPPA) test (FUJIREBIO INC., Tokyo, Japan), following the manufacturer’s instructions. All reactive TRUST samples were titrated by serial dilutions. CSF specimens undergone VDRL testing (Becton, Dickinson and Company, Franklin Lakes, NJ, USA), TRUST testing, white blood cell (WBC) counting and protein concentration assessment. CSF-TPPA was considered reactive if the titer was ≥ 1:80. All reactive CSF-TPPA samples were titrated by serial dilutions.

### 2.4 Statistical analysis

Statistical analyses were performed by SPSS software (version 30.0; Chicago, IL, USA) and R software (version 4.4.1, R Project for Statistical Computing). Reciprocal and log10 transformation were applied to titer data of serum TRUST and CSF-VDRL and CSF-TPPA. Continuous variables were presented as median (interquartile range), while categorical variables were presented as n (%). Mann-Whitney U-tests were used to comparing continuous variables with nonparametric data, and Chi-squared tests were applied to compare categorical variables. Spearman’s rank correlation was used to analyses the correlation between variables. Receiver operating characteristic (ROC) analysis was used to assess the performance of CSF-TPPA titer, with the optimal cut-off determined by the maximal Youden’s index (sensitivity+specificity-100%). Positive predictive values (PPVs) and negative predictive values (NPVs) were calculated using standard formulae. ROC analysis was performed using Medcal V.23.0.1 (Broekstraat, Belgium). Restricted cubic spline (RCS) logistic regression was used to explore the non-linear relationship between CSF-TPPA titer and neurosyphilis. Preliminary investigation suggested that three knots should be used in the restricted cubic spline model for CSF-TPPA titer. We adjusted the splines for patient gender, age, syphilis stage, prior syphilis treatment, neuropsychiatric symptoms/signs and serum TRUST titer. The logistic regression model was used to estimate the odds ratio (OR) for neurosyphilis, with an OR of 1 as the reference value. Factors with a p value < 0.05 were considered statistically significant.

## 3 Results

### 3.1 Clinical characteristics

**Figure 1:**
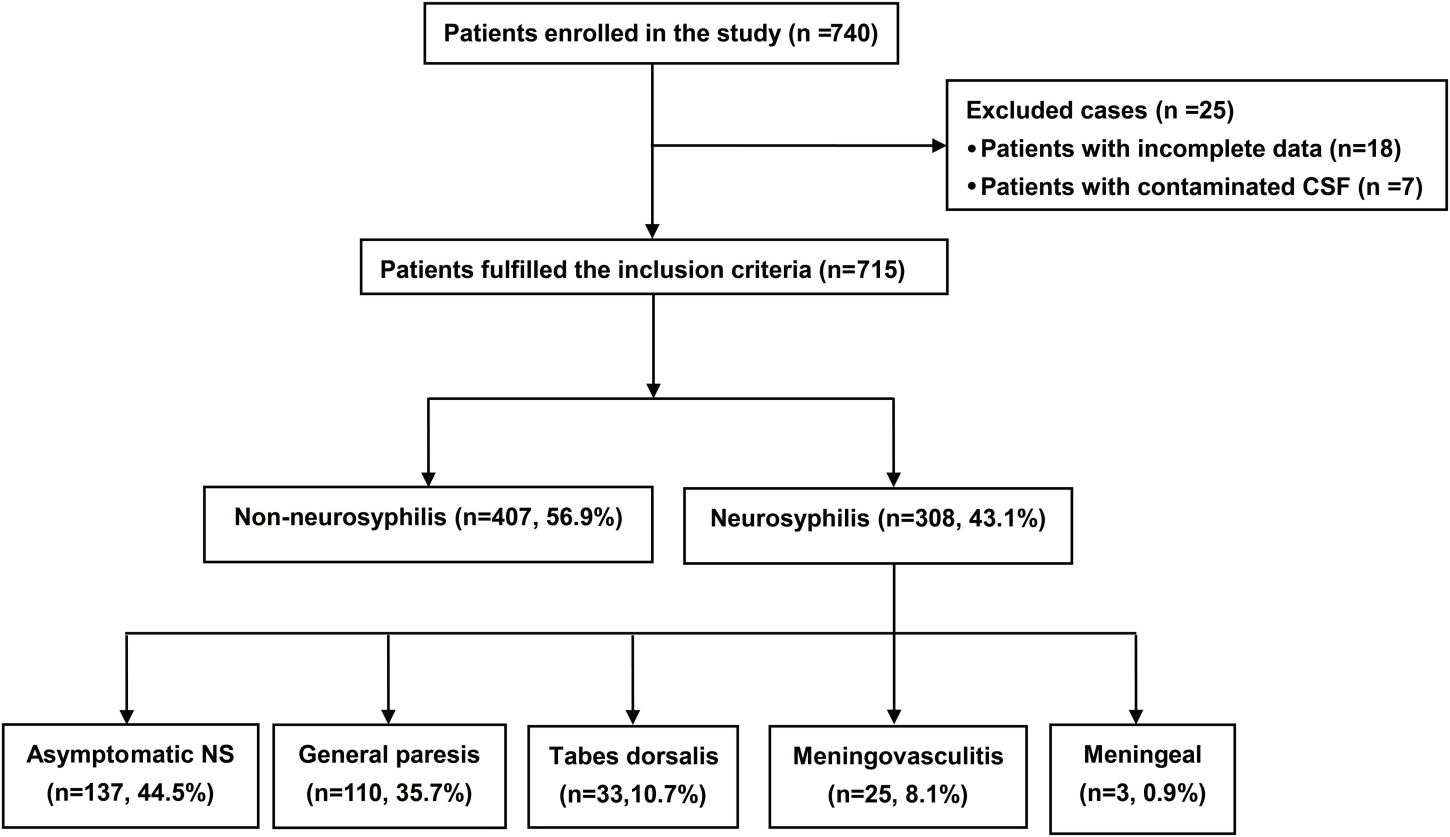
Enrollment flow chart of patients. CSF, cerebrospinal fluid; NS, neurosyphilis

Between April 2020 and January 2022, 715 eligible patients were included in this analysis. The stage and clinical spectrum of the patients were showed in **Figure 1**. The overall incidence of neurosyphilis was 43.1% (308/715). Among those diagnosed with neurosyphilis, 137 (44.5%) were with asymptomatic neurosyphilis and 171 (55.5%) had symptomatic neurosyphilis. General paresis account for a highest proportion (110 cases, 35.7%) among symptomatic neurosyphilis patients, followed by tabes dorsalis (33 cases, 10.7%), meningovasculitis (25 cases, 8.1%), and meningeal syphilis (3 cases, 0.9%). Additionally, 5 cases were complicated by ocular syphilis and 3 by otosyphilis. The clinical features, serological results and CSF assessment of the 715 included patients are summarized in **Table 1**. The median serum TRUST titer was 1:16 (IQR, 1:8 to 1:32). CSF-VDRL was positive in 269 cases (37.6%), and the median titer of CSF-VDRL was 1:2 (IQR, 1:1 to 1:8) in patients with neurosyphilis. CSF- TRUST was positive in 224 cases (31.3%), and the median titer of CSF-TRUST was 1:2 (IQR, Neg to 1:4) in patients with neurosyphilis. Additionally, 22.4% of patients had a CSF-WBC count greater than 10 cells/μL, and 34.1% had a CSF-protein concentration higher than 0.5g/L.

**Table 1:** Clinical features, serological and CSF assessment results of 715 included patients.

Characteristic	Total (n=715)	NS (n=308)	N-NS (n=407)	*p* value
Gender (n, %)				<0.001
Female	304 (42.5%)	77 (25.0%)	227 (55.8%)	
Male	411 (57.5%)	231 (75.0%)	180 (44.2%)	
Age (yr, median [IQR])	52.0 [34.0; 62.0]	57.0 [49.0; 64.0]	41.0 [29.0; 58.0]	<0.001
Syphilis stage (n, %) ^a^				<0.001
Early stage	82 (11.5%)	10 (3.2%)	72 (17.7%)	
Late stage	633 (88.5%)	298 (96.8%)	335 (82.3%)	
Prior syphilis treatment (n, %) ^b^				0.144
Yes	497 (69.5%)	223 (72.4%)	274 (67.3%)	
No	218 (30.5%)	85 (27.6%)	133 (32.7%)	
With neuropsychiatric symptoms/signs (n, %)				<0.001
Negative	514 (71.9%)	136 (44.2%)	378 (92.9%)	
Positive	201 (28.1%)	172 (55.8%)	29 (7.1%)	
Serum TRUST titers				
Median [IQR]	1:16 [1:8; 1:32]	1:32 [1:8; 1:64]	1:16 [1:4; 1:32]	<0.001
CSF-VDRL titers				
Median [IQR]	Neg [Neg; 1:2]	1:2 [1:1; 1:8]	Neg [Neg; Neg]	<0.001
Positive rate (n, %)	269 (37.6%)	269 (87.3%)	0 (0.0%)	
CSF WBC (cells/ul)				
Median [IQR]	4.0 [2.0; 10.0]	10.0 [4.7; 35.8]	2.0 [0.0; 6.0]	<0.001
CSF protein (g/l)				
Median [IQR]	0.4 [0.29; 0.58]	0.62 [0.46; 0.81]	0.31 [0.24; 0.40]	<0.001
CSF-TRUST titers				
Median [IQR]	Neg [Neg; 1:1]	1:2 [Neg; 1:4]	Neg [Neg; Neg]	<0.001
Positive rate (n, %)	224 (31.3%)	224 (65.9%)	0 (0.0%)	
CSF TPPA titers				
Median [IQR]	1:160 [Neg; 1:2560]	1:2560 [1: 1280; 1:10240]	Neg [Neg; Neg]	<0.001
Positive rate (n, %)	443 (62.0%)	308 (100%)	135 (33.2%)	<0.001

a Early syphilis included primary, secondary, and early latent syphilis; late syphilis included late latent syphilis, latent syphilis of unknown duration, and tertiary syphilis.

b Patients have received benzathine penicillin for syphilis before, but not for neurosyphilis, which do not reach treponemacidal levels in the central nervous system. NS, neurosyphilis; N-NS, non-neurosyphilis; IQR, interquartile range; CSF, cerebrospinal fluid; TRUST, toluidine red unheated serum test; VDRL, Venereal Disease Research Laboratory; Neg, negative; WBC, white blood cell; TPPA, Treponema pallidum particle agglutination.

The proportion of male patients in the neurosyphilis (NS) group was higher than that in the non-neurosyphilis(N-NS) group (75.0% versus 44.2%, *p* < 0.001). The median age of NS group was higher than the N-NS group (57.0 years old versus 41.0 years old, *p* < 0.001). There was a statistically significant association between NS and late stage of syphilis (*p* < 0.001). More patients (55.8%) in the NS group had neurological symptoms than patients (7.1%) in the N-NS group (*p* < 0.001). Patients in the NS group had significantly higher serum TRUST titer (median, 1:32 versus 1:16, *p* < 0.001) than patients in the N-NS group (**Table 1**).

### 3.2 Performance of CSF-TPPA in neurosyphilis vs. non-neurosyphilis

CSF-TPPA was reactive in 443 cases (62.0%), with a median titer of 1:160 (IQR, Negative to 1:2560). All the patients with a reactive CSF-VDRL also had a reactive CSF-TPPA result. Patients in the NS group exhibited significantly higher CSF-TPPA titer (median, 1:2560 vs negative, p < 0.001) compared to the N-NS group (**Table 1**).

**Figure 2:**
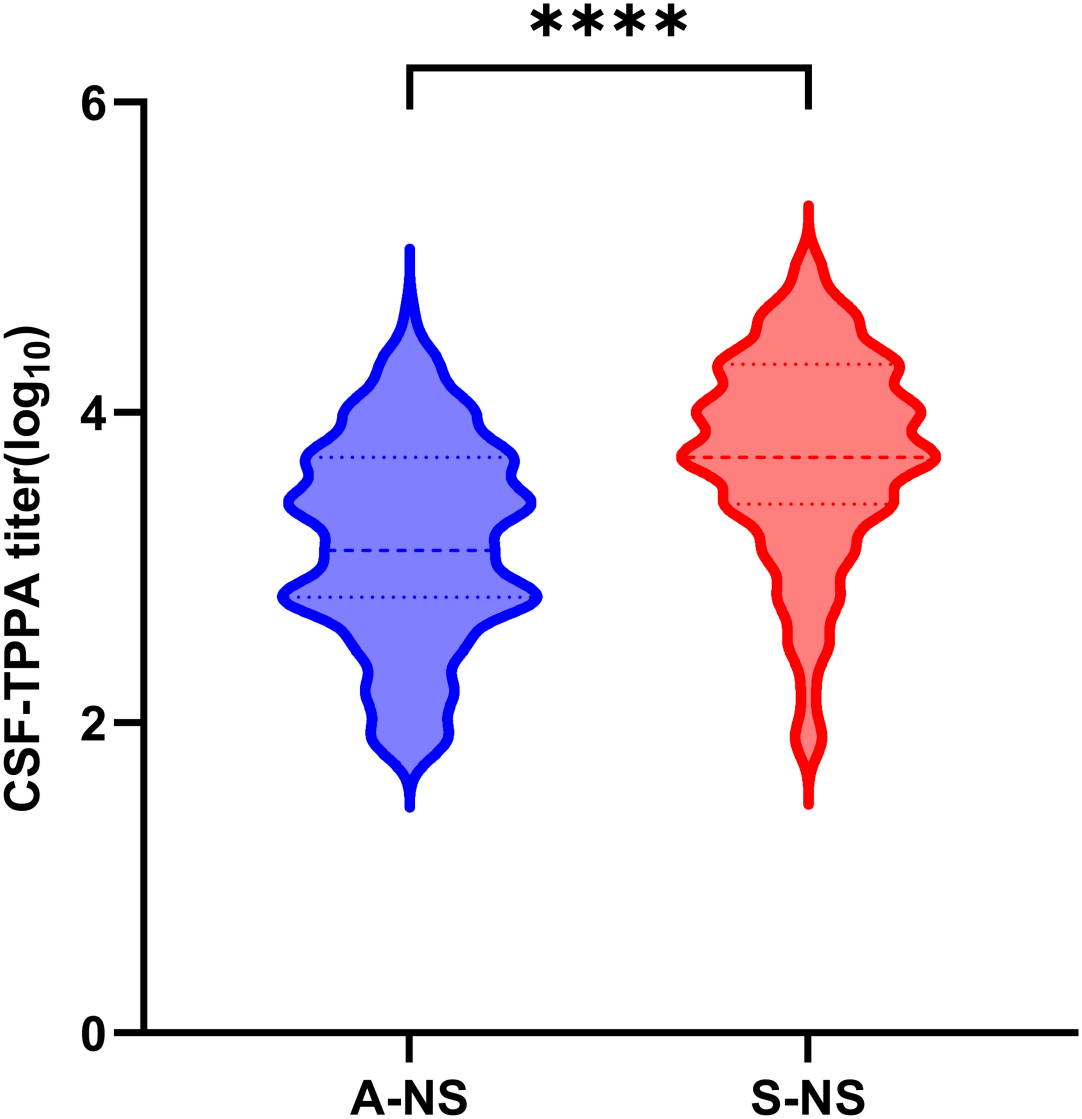
Levels of CSF-TPPA titer in the A-NS and S-NS group. A-NS, asymptomatic neurosyphilis; S-NS, symptomatic neurosyphilis. ****p > 0.0001.

The median CSF-TPPA titer in symptomatic neurosyphilis patients was more than four times higher than that in asymptomatic neurosyphilis patients (median, 1:5120 vs 1:1280, p < 0.0001) (**Figure 2**).

### 3.3 Diagnostic value of CSF-TPPA titer for neurosyphilis

**Figure 3:**
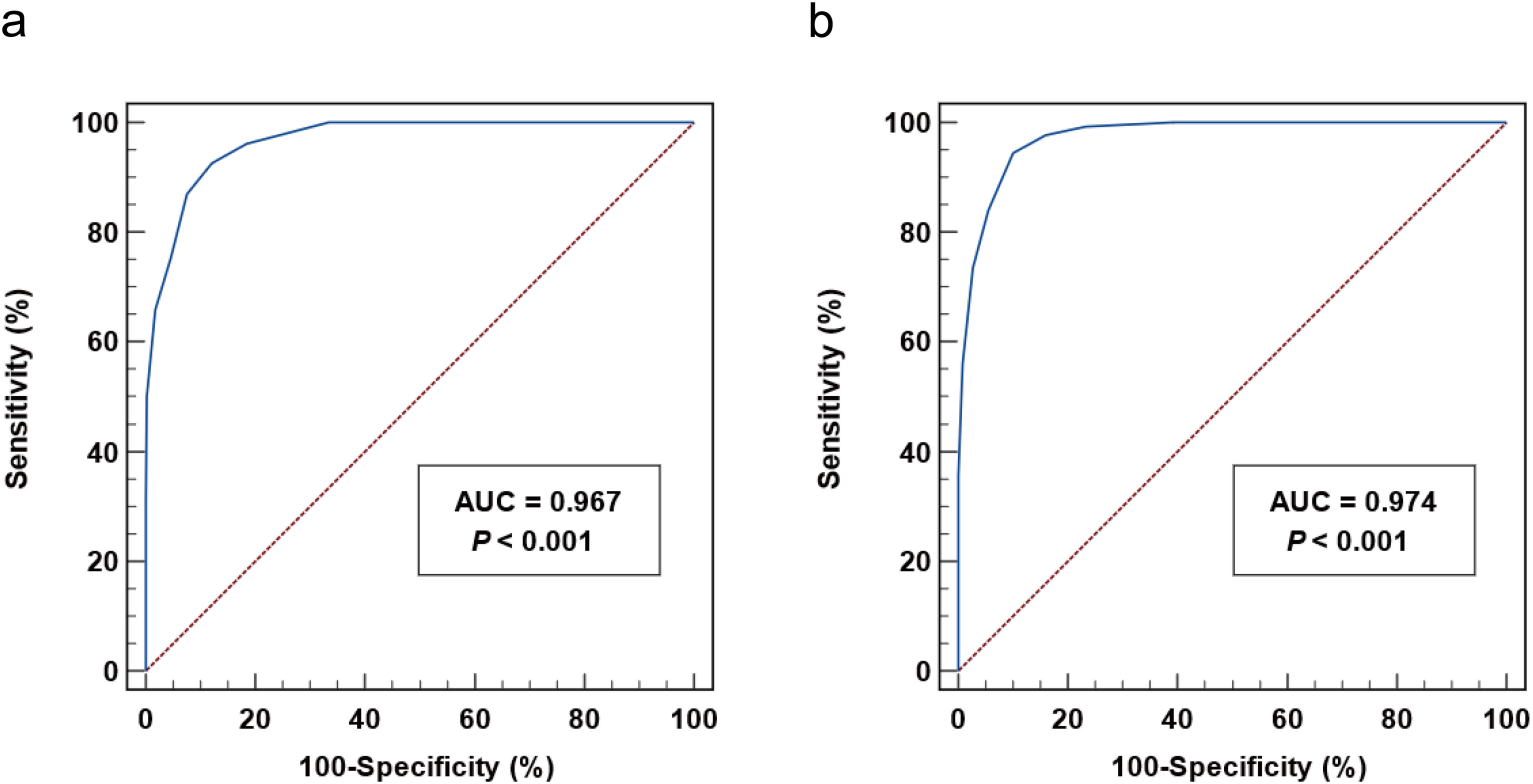
Receiver operating characteristic curve analysis of CSF-TPPA titers in the diagnosis of NS. **(a)** The diagnostic performance of CSF-TPPA titer for neurosyphilis. **(b)** The diagnostic performance of CSF-TPPA titer for reactive CSF VDRL. CSF, cerebrospinal fluid; TPPA, Treponema pallidum particle agglutination; AUC, area under curve.

The CSF-TPPA titer was significantly correlated with CSF-VDRL (*r*_s_=0.819, p < 0.001), CSF WBC count (*r*_s_=0.521, *p* < 0.001) and CSF protein levels (*r*_s_=0.638, p < 0.001), suggesting its potential utility for NS diagnosis (**Table 2**). ROC analyses were conducted to evaluate the diagnostic accuracy of CSF-TPPA titers in neurosyphilis. The area under curve (AUC) for CSF-TPPA was 0.967 (95% confidence interval (CI): 0.951- 0.979), with an optimal cut-off titer of ≥ 1:320. This threshold provided a sensitivity of 92.53%, specificity of 87.96%, PPV of 85.33%, and NPV of 93.96% (**Figure 3a**). The diagnostic performance (specificity, sensitivity, PPV and NPV) of CSF-VDRL, CSF-TRUST, CSF-TPPA and CSF-TPPA titer ≥ 1:320 for neurosyphilis was summarized in **Table 3**.

**Table 2:** Correlation between CSF TPPA titers and CSF parameters.

	CSF-VDRL titer		CSF WBC		CSF protein	
	*r_s_*	*p* value	*r_s_*	*p* value	*r_s_*	*p* value
CSF-TPPA titer	0.819	<0.001	0.521	<0.001	0.638	<0.001

Correlation was evaluated using Spearman’s rank correlation. CSF, cerebrospinal fluid; VDRL, Venereal Disease Research Laboratory; WBC, white blood cell; TPPA, Treponema pallidum particle agglutination; *r_s_*, Spearman rank correlation coefficient.

**Table 3:** Specificity, sensitivity, PPV and NPV of CSF Parameters for neurosyphilis diagnosis.

	%Sensitivity (95% CI)	%Specificity (95% CI)	%PPV (95% CI)	%NPV (95% CI)
CSF-VDRL	87.33 (82.98- 90.74)	100 (98.83-100)	100 (98.25-100)	91.26 (88.14-93.63)
CSF-TRUST	72.72 (67.33-77.55)	100 (98.83-100)	100 (97.90-100)	82.89 (79.20-86.06)
CSF-TPPA	100 (98.46-100)	66.83 (61.99-71.35)	69.53 (64.97-73.74)	100 (98.26-100)
CSF-TPPA titer ≥ 1:320	92.53 (88.86-95.10)	87.96 (84.30- 90.88)	85.33 (80.97- 88.85)	93.96 (90.95- 96.05)

CI, confidence interval; PPV, positive predictive value; NPV, negative predictive value; CSF, cerebrospinal fluid; VDRL, Venereal Disease Research Laboratory; TRUST, toluidine red unheated serum test; TPPA, Treponema pallidum particle agglutination.

When using CSF-VDRL as the standard test for confirmed neurosyphilis, the AUC for CSF-TPPA was 0.974 (95% CI:0.965-0.983). The optimal cut-off titer for confirmed neurosyphilis was ≥ 1:640, yielding a sensitivity of 94.42%, specificity of 89.91%, PPV of 84.95%%, and NPV of 96.39%% (**Figure 3b**). At the threshold of CSF-TPPA ≥ 1:320, the sensitivity for confirmed neurosyphilis diagnosis was 97.77%, with a specificity of 84.08%.

Among the cohort, 71 cases (9.9%) with non-reactive CSF-VDRL presented with elevated CSF WBC count (>10/μL) or elevated CSF protein concentration (>0.5g/L). Of those, 39 cases (54.9%) had reactive CSF-TPPA results, and 22 cases (31.0%) had CSF-TPPA ≥ 1:320. Using reactive CSF-TPPA combined with elevated CSF-WBC counts or CSF-protein concentrations as diagnostic criteria for presumptive neurosyphilis, the sensitivity and specificity of CSF-TPPA ≥ 1:320 were 56.41% (95%CI:39.76-71.81%) and 100% (95%CI:86.66-100.100%) respectively in these cases. Notably, 10 cases with neuropsychiatric signs or symptoms, including one case with vision loss, had nonreactive CSF-VDRL results but exhibited elevated CSF WBC counts or CSF protein levels. Among these, five cases were consistent with symptomatic neurosyphilis (two cases with general paresis; two cases with tabes dorsalis; one case with optic atrophy). CSF-TPPA titers in these cases ranged from 1:320-1:5120. The remaining five cases were differentially diagnosed with vascular dementia or cerebral infarction, two had CSF-TPPA titers equal to 1:80, while three had non-reactive CSF-TPPA results.

### 3.4 Restricted cubic spline (RCS) curves analysis

**Figure 4:**
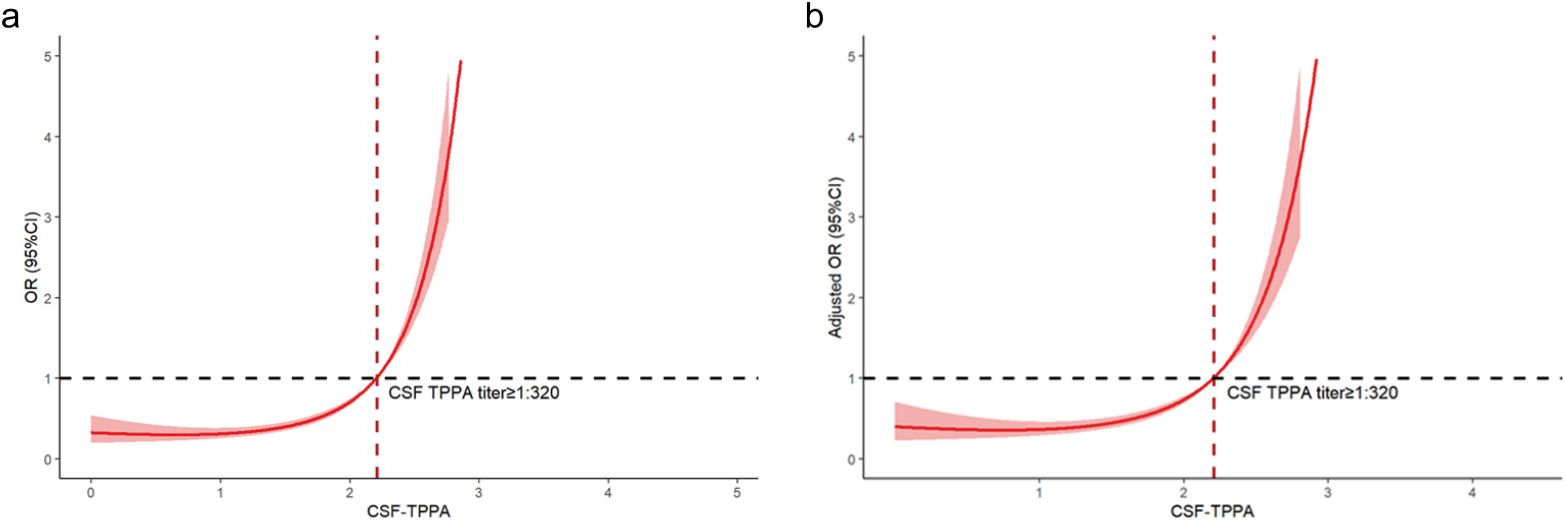
Restricted cubic spline of the association between CSF-TPPA titer and neurosyphilis. **(a)** Unadjusted association between CSF-TPPA titer and neurosyphilis. **(b)** Gender, age, syphilis stage, prior syphilis treatment, neuropsychiatric symptoms/signs, serum TRUST titer were adjusted in the restricted cubic spline. CSF, cerebrospinal fluid; TPPA, Treponema pallidum particle agglutination.

**Figure 4** illustrates the restricted cubic spline curves, showing the relation between CSF-TPPA titer and neurosyphilis. A nonlinear relation was observed between CSF-TPPA titer and neurosyphilis (*p* for nonlinearity < 0.0001) (**Figure 4a**). The OR for the association increased with higher CSF-TPPA titers, and when CSF-TPPA titers reached ≥ 1:320, the OR was significantly higher than 1. In **Figure 4b**, we used RCS analysis to visualize the relation of CSF-TPPA titer and neurosyphilis after adjusted for gender, age, syphilis stage, prior syphilis treatment, neuropsychiatric symptoms/signs, and serum TRUST titers. Regarding the strong “J-shaped” associations relation between CSF-TPPA titers and neurosyphilis, the plot showed a substantial reduction of the risk within the lower range of CSF-TPPA titer, which was relatively flat until CSF-TPPA ≥ 1:320 and then escalated rapidly (*p* for non-linearity <0.0001).

## 4 Discussion

The diagnosis of neurosyphilis is inherently complex, given the lack of a definitive and universally reliable laboratory test. CSF-VDRL remains the gold standard, but its low sensitivity often leads to missed diagnoses. This diagnostic gap necessitates the exploration of alternative tools, particularly for cases of high clinical suspicion but negative CSF-VDRL results. Our study systematically evaluates the diagnostic performance of three CSF-based tests—CSF-TRUST, CSF-VDRL and CSF-TPPA—and demonstrates the promising role of CSF-TPPA titer as a sensitive and practical marker for neurosyphilis.

Among the three tests evaluated, CSF-TRUST demonstrated comparable specificity to CSF-VDRL but lower sensitivity (72.72% vs. 87.33%). In contrast, CSF-TPPA exhibited the highest sensitivity (100%) but the lowest specificity (66.83%). These findings align with the well-established understanding of the respective roles of these tests: CSF-TRUST and CSF-VDRL provide high specificity, whereas CSF-TPPA excels in sensitivity. Importantly, CSF-TPPA offers additional practical advantages, as it requires no specialized equipment, making it accessible for resource-limited settings.

The diagnostic value of quantifying CSF-TPPA titers has been relatively underexplored. In our study, correlation analysis revealed that CSF-TPPA titer was strongly associated with CSF-VDRL titer and CSF-protein levels, while demonstrating moderate correlation with CSF -WBC counts. ROC analysis indicated that CSF-TPPA titer has excellent diagnostic accuracy, with an AUC of 0.967. A threshold of CSF-TPPA titer ≥ 1:320 significantly improved specificity (87.96% vs. 66.83%) compared to the unquantified CSF-TPPA test while maintaining high sensitivity of 92.53%.

Similar results have been reported in the literature. For instance, Lu et al. (14). identified a CSF-TPPA titer ≥ 1:320 as an optimal cutoff with a sensitivity of 90.00% and specificity of 84.00%, closely mirroring our findings. Similarly, another study reported sensitivities and specificities of 84.09% and 88.89%, respectively, for the same threshold (15). These studies underscore the reproducibility and reliability of the CSF-TPPA titer ≥ 1:320 cutoff. Furthermore, Marra et al. (11) and the UK national guidelines (12) advocate for higher thresholds (CSF-TPPA titer ≥ 1:640) to maximize specificity under certain diagnostic criteria. Our study corroborates these higher thresholds, demonstrating that CSF-TPPA titer ≥ 1:640 achieves a sensitivity of 94.42% and specificity of 89.91% according to the neurosyphilis definition of reactive CSF-VDRL.

In our study, symptomatic neurosyphilis patients exhibited significantly higher CSF-TPPA titer than asymptomatic neurosyphilis patients. For those presumptive neurosyphilis patients, the sensitivity of CSF-TPPA ≥1:320 was relatively low. It was noticed that CSF-TPPA ≥1:320 could effectively identified symptomatic neurosyphilis for those cases with nonreactive CSF-VDRL results but elevated CSF WBC counts or CSF protein levels.

An innovative aspect of this study was the application of RCS logistic regression to examine the nonlinear relationship between CSF-TPPA titer and neurosyphilis risk. This approach revealed a key inflection point at a CSF-TPPA titer of 1:320, after which the odds of neurosyphilis increased sharply. The RCS analysis complements ROC findings by offering a nuanced understanding of how CSF-TPPA titer correlates with neurosyphilis risk across a continuous range. Notably, when CSF-TPPA titer exceeded 1:320, the odds ratio for neurosyphilis rose significantly, reinforcing the diagnostic relevance of this threshold.

Previous studies have highlighted various predictors of neurosyphilis, including gender (16–18), age (16, 19), syphilis stage (20, 21), treatment history (20, 22), neuropsychiatric symptoms (23, 24) and serum TRUST/RPR titer (20, 25). Our study uniquely adjusted for these confounding factors in the RCS logistic regression model. Even after full adjustment, the association between CSF-TPPA titer and neurosyphilis remained robust, with the prevalence of neurosyphilis increasing sharply beyond a CSF-TPPA titer of 1:320. This reinforces the independent diagnostic value of CSF-TPPA titer as a biomarker for neurosyphilis.

The findings of this study have significant clinical implications. First, the high sensitivity of CSF-TPPA ensures that it can reliably exclude neurosyphilis when negative. Second, the identification of CSF-TPPA titer thresholds (≥1:320 and ≥1:640) provides clinicians with practical benchmarks for diagnosis, particularly in cases when CSF-VDRL is negative. Finally, the straightforward methodology and equipment-free nature of CSF-TPPA testing make it an attractive diagnostic tool for resource-limited settings.

Despite its strengths, our study has limitations. The CSF-TPPA titer cutoff of ≥1:320 identified in this study requires further validation in independent, multi-center cohorts with varied demographic and clinical characteristics. Such efforts would help establish its universal applicability and refine potential geographic or pathogen-specific variations. The cross-sectional design precludes the assessment of CSF-TPPA titer dynamics over time, such as its potential role in monitoring treatment response. Additionally, our findings are based on a cohort of HIV-negative patients, limiting generalizability to HIV-positive populations. Future studies should explore the diagnostic and prognostic utility of CSF-TPPA titer in diverse populations and evaluate its role in guiding neurosyphilis treatment.

In conclusion, CSF-TPPA titer is a highly sensitive and practical marker for diagnosing neurosyphilis. A titer threshold of ≥1:320 significantly enhances specificity without compromising sensitivity, offering a reliable alternative in cases where CSF-VDRL is negative. The integration of CSF-TPPA titer into clinical practice has the potential to address critical gaps in neurosyphilis diagnostics and improve patient outcomes. Future research should continue to refine the diagnostic criteria and explore the broader applicability of this promising biomarker.

## Data Availability

The raw data supporting the conclusions of this article will be made available by the authors, without undue reservation.
